# Online Measurement of Soil Organic Carbon as Correlated with Wheat Normalised Difference Vegetation Index in a Vertisol Field

**DOI:** 10.1155/2014/569057

**Published:** 2014-07-03

**Authors:** Yücel Tekin, Yahya Ulusoy, Zeynal Tümsavaş, Abdul M. Mouazen

**Affiliations:** ^1^Vocational School of Technical Science, Uludag University, 16059 Bursa, Turkey; ^2^Agricultural Faculty, Uludag University, 16059 Bursa, Turkey; ^3^Environmental Science and Technology Department, Cranfield University, Bedfordshire MK43 0AL, UK

## Abstract

This study explores the potential of visible and near infrared (vis-NIR) spectroscopy for online measurement of soil organic carbon (SOC). It also attempts to explore correlations and similarities between the spatial distribution of SOC and normalized differential vegetation index (NDVI) of a wheat crop. The online measurement was carried out in a clay vertisol field covering 10 ha of area in Karacabey, Bursa, Turkey. Kappa statistics were carried out between different SOC and NDVI data to investigate potential similarities. Calibration model of SOC in full cross-validationresulted in a good accuracy (*R* ^2^ = 0.75, root mean squares error of prediction (RMSEP) = 0.17%, and ratio of prediction deviation (RPD) = 1.81). The validation of the calibration model using laboratory spectra provided comparatively better prediction accuracy (*R* ^2^ = 0.70, RMSEP = 0.15%, and RPD = 1.78), as compared to the online measured spectra (*R* ^2^ = 0.60, RMSEP = 0.20%, and RPD = 1.41). Although visual similarity was clear, low similarity indicated by a low Kappa value of 0.259 was observed between the online vis-NIR predicted full-point (based on all points measured in the field, e.g., 6486 points) map of SOC and NDVI map.

## 1. Introduction

Soil organic carbon (SOC), the major component of soil organic matter, is extremely important for land use and management. Agricultural management of land plays an important role in global warming mitigation due to its effects on SOC dynamics [1]. Many management practices that are effective in increasing SOC are also advantageous in increasing aggregate stability, enhancing soil fertility, and improving crop yield. It is achieved by adding organic materials, composts, manure, and other recycled organic materials to the soil. A method to map the spatial variability of SOC would be a very useful tool to optimize the spatial distribution of artificially added SOC.

Proximal and remote sensors are being increasingly used in agriculture to control and manage farming inputs. For example, they are extensively used in precision agriculture (PA) in order to identify proper targets and needs of crops for variable rate applications [2]. However, the main requirement, for these sensors, is their robustness and more importantly they must provide accurate and meaningful data. One of the most rapid and promising measurement techniques for PA applications is the visible and near infrared (vis-NIR) spectroscopy. It is a simple and nondestructive analytical method that can be used to enhance, complement, or replace conventional methods of soil analyses. It is particularly useful to overcome some of the limitations of conventional laboratory methods and may be utilized to predict several soil properties simultaneously [3]. Vis-NIR spectroscopy has become the most attractive technique for end-users of PA, as some recent studies by Mouazen et al. [4], Viscarra-Rossel and Chen [5], Tekin et al. [6], and Kodaira and Shibusawa [7] prove it to provide accurate quantification of main physical and chemical soil properties that is useful for digital soil mapping.

Many researchers have successfully measured SOC by using the vis-NIR spectroscopy [4, 8–13]. Stenberg et al. [14] provided a comprehensive analysis of the literature that confirmed the possibility of successful measurement of SOC with vis-NIR, which can be attributed to the direct spectral response of soil carbon in the NIR range. Vis-NIR spectra of soils contain large sets of spectral variables, which upon being modeled with linear algorithms compensate only partly negative effects such as collinearity, noise, and reduction of dimensionality [15]. The complex relationship between spectral signatures and the soil property can be better modeled by multivariate regression methods, which have an advantage over simple bivariate relationships based on, for example, peak intensity measurements [16]. Partial lease squares (PLS) regression is the most common technique adopted today to model the relationships between infrared spectral intensities characteristics of the soil components and the soil properties through derived PLS loadings, scores, and regression coefficients [17]. PLS regression establishes a series of components or latent vectors that provide a simultaneous reduction or decomposition of *X* and *Y* such that these components explain, as much as possible, the covariance between *X* and *Y* [18]. One of the advantages of PLS regression compared to other chemometric methods, for example, principal component regression analysis, is the possibility to interpret the first few latent variables, because they show the correlations between the property values and the spectral features [19]. Several researchers have proved PLS regression resulting in excellent prediction performance of SOC [10, 20]. Literature also documents success in online (tractor mounted) measurement of SOC using the PLS, which has the advantage of providing high resolution data and allows mapping of the spatial variability of soil properties [4, 21].

Total standing biomass or vegetative cover reflects total ecosystem productivity and is often proportional to the C and N input to the soil [22]. Active canopy sensors provide a relative measure of crop N status and variation of crop density, which can be attributed to the soil properties. Crop canopy sensors are relatively small in size and operate by directing sensor produced visible (VIS) and near infrared (NIR) light at the plant canopy and recording the amount of VIS and NIR light that is reflected [23]. The measurement of variation of crop density is defined by normalized differential vegetation index (NDVI) or leaf area index (LAI). NDVI is related to amount of photosynthetically active absorber by the canopy maps. NDVI can be used to interpret spatial patterns of pest and disease infestation, water status, crop characteristics, and quality [24]. It was also used to inform variable rate nitrogen fertilization [25–29]. There are attempts to correlate NDVI with soil characteristics measured with slow, expensive, and tedious traditional laboratory analytical methods, which allow only limited number of readings [30]. However, to the best of our knowledge, there is no study about establishing correlations between NDVI and soil fertility indicators (e.g., SOC), measured with online soil sensors.

The aim of this study is to explore the potential of a vis-NIR online sensor to measure SOC and to establish correlations with NDVI data measured for wheat in a field with vertisol in Bursa region in Turkey.

## 2. Materials and Methods

### 2.1. Online Soil Sensor

**Figure 1:**
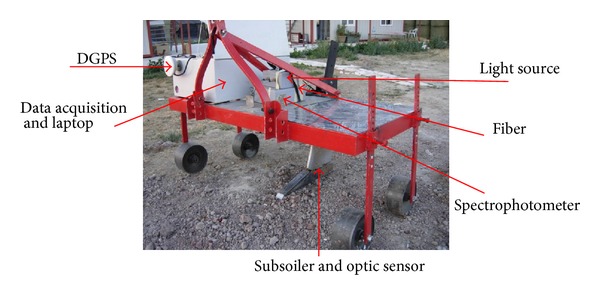
The online visible and near infrared (vis-NIR) soil sensor attached to the three-point linkage of a tractor, simulating the design of Mouazen et al. [31].

The online soil sensor consists of a subsoiler that penetrates the soil to any depth between 5 and 40 cm depth, making a trench in the soil, whose bottom is smoothened due to the downward forces acting on the subsoiler. The optical unit is attached to the backside of the subsoiler chisel to acquire soil spectra from the smooth bottom of the trench in diffuse reflectance mode. The subsoiler and the optical unit are attached to a metal frame, which is mounted onto the three-point linkage of a tractor [4]. The metal frame of the sensor has been manufactured in Uludag University using the same design of Mouazen [31] (Figure 1). During field measurement the online sensor was set at 15 cm deep and driven at a moving speed of approximately 3 km h^−1^.

To measure soil spectra, AgroSpec mobile, fiber type, vis-NIR spectrophotometer (Tec5 Technology for Spectroscopy, Germany) was used. The measurement range was of 305–2200 nm. A differential global positioning system (DGPS) (EZ-Guide 250, Trimble, USA) was used to record the position of the online measured spectra with <1 meter accuracy. The spectrophotometer, light source, DGPS, and laptop of Cranfield University were set up on the newly manufactured frame. The frame and the online sensor were tested in Uludag University farm before the actual field measurements to avoid unexpected malfunction of both software and cable connections during field measurement. The AgroSpec software that serves as the platform for the mobile spectrometer system was used to acquire data. This software is specially designed to meet the requirements of agriculture, ecology, and geoscience applications.

### 2.2. Canopy Sensor

**Figure 2:**
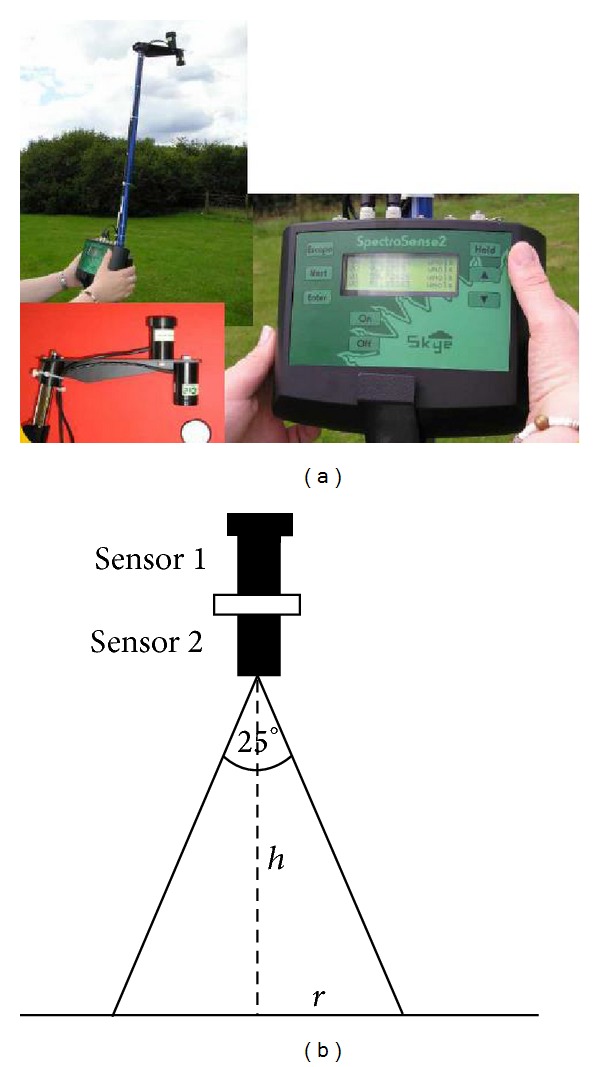
SpectroSense SKL925 canopy sensor and area of measurement.

The canopy sensor SpectroSense SKL925 (SKYE, UK) was used to measure the crop (wheat) NDVI (Figure 2). Vegetation indices can be calculated as the ratios of different wavebands of reflected solar radiation and are related to the abundance and activity of radiation absorbers such as water and plant chlorophyll. The sensor is fitted with a removable cosine correcting light acceptance head. When taking incident or downwelling light measurements, the head is left in place so that the sensor is fully cosine corrected (accepts light in accordance with Lambert's Cosine Law). Sensor 1 is fitted with the cosine correcting head to measure incident light. Sensor 2 is of a narrow angle and measures reflected light (Figure 2). Both incident and reflected light are measured simultaneously by 2 identical sensors, to eliminate fluctuations in solar radiation. Without the cosine head, both 2 and 4 channel sensors have a 25° cone field of view (12.5° off perpendicular). The area of ground in view to the sensor is then defined by the height above the ground, as shown in Figure 2.

NDVI is a normalized ratio of Red (R) and NIR, as defined by (1). NDVI values range from −1 to +1, where negative values generally indicate low vegetation or low canopy, and +1 value is indicative of the highest possible density of green leaves or canopy:

(1) $$\begin{array}{c} \text{NDVI}=\frac{\left(Z*800_{\text{R}(\text{nA})}*Y\right)-\left(650_{\text{R}(\text{nA})}*X\right)}{\left(Z*800_{\text{R}(\text{nA})}*Y\right)+\left(650_{\text{R}(\text{nA})}*X\right)}, \end{array}$$

where *X* is NIR_I_ incident reading (in *μ*mol·m^−2^·s^−1^), *Y* is Red_I_ incident reading (in *μ*mol·m^−2^·s^−1^), *Z* is ratio sensitivity of reflected NIR and Red, NIR_R(nA)_ is reflected reading in nanoamps (or direct current output), and Red_R(nA)_ is reflected reading in nanoamps (or direct current output).

### 2.3. Experimental Site and Measurement

**Figure 3:**
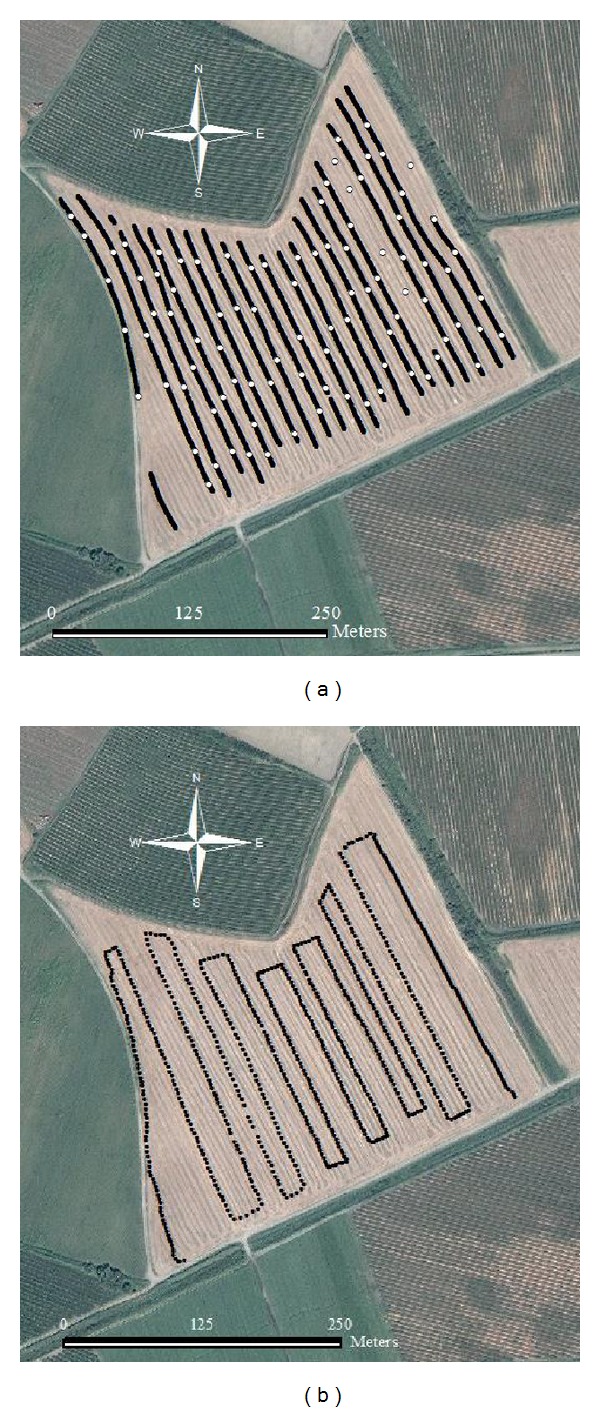
Measured transects with soil sampling positions for the online soil measurement (a). Measured transect for the NDVI measurement (b).

The field was of 10 ha area located in Karacabey in the Karaca Farm in Bursa, Turkey. The field was of a clay soil (Table 1) and of minor slope variation. The canopy sensor measurements were taken on May 24, 2013, whereas the online soil measurements were taken on June 25, 2013, after crop harvest. Canopy spectral reflectance was measured using SKYE handheld optical sensor from 10:00 a.m. to 12:00 a.m. according to local time under cloudless conditions at a height (*h*) 0.75 m above the wheat canopy, for which a radius (*r*) of 0.17 m and area of 0.09 m^2^ were targeted. The logging interval was 5 seconds during NDVI measurements. NDVI data were collected from 925 points covering the entire field. Locations of these points were recorded with a GPS (Figure 3).

The online soil measurements were carried out in evening time due to high temperatures (>35°C). The online vis-NIR sensor scanned 14 adjacent lines at 20 m intervals at a moving speed of 3 km h^−1^. A total of 92 bulk soil samples were collected from the bottom of the furrow. The sampling positions were recorded with the DGPS. Sampling lines and sampling positions are shown in Figure 3.

The 92 soil samples collected during the online measurement were used for calibration and validation. The samples were equally divided into two parts. First half was used for laboratory reference measurements of SOC, moisture content (MC), and particle size distribution (PSD), and the other half was used for optical scanning in the laboratory. SOC was measured with help of the Walkley-Black method [32]. The PSD was measured by sieving and sedimentation method [33]. Soil MC was measured by oven drying of samples at 105° for 24 h [34]. The entire set of 92 samples was used for SOC and MC analyses, whereas only 19 selected samples were used for PSD analyses. PSD and MC results are shown in Table 1. Measured maximum, minimum, and mean SOC values of the field were relatively small by 1.93%, 0.81%, and 1.41%, respectively, with small variability (SD = 0.22%). This may indicate potential difficulties in obtaining successful correlations with soil spectra, as the smaller is the field variability of a soil property, the smaller is the chance for obtaining good calibration models [35].

### 2.4. Optical Measurement in the Laboratory

The 92 soil samples collected during the online measurements were scanned in laboratory using the same vis-NIR spectrophotometer (AgroSpec, tec5 Technology for Spectroscopy, Germany), used during the online field measurement. Before scanning, plant material and stones were removed and each sample was mixed. Then each soil sample was placed into three plastic cups having 1.2 cm depth and 1.2 cm diameter. The soil in the cup was carefully pressed and leveled to form a smooth scanning surface [36]. A 100% white reference was scanned before soil scanning and was repeated after every 30 minutes. Each cup was scanned 10 times, and obtained readings were averaged to yield the spectrum for the cup. The final spectrum, used for further analysis, was an average of the three spectra obtained for the three cups.

### 2.5. Model Establishment and Validation

Since the number of soil samples collected in the field was relatively small to build a field scale calibration, 324 external soil samples collected from other fields across Europe were used. These samples were divided as follows: 147 samples were collected from Vindumovergaard Farm (Denmark), 82 samples from Duck End farm (UK), 21 samples from Shrewsbury field (UK), 34 samples from Ten Acre Meadow Farm (UK), 16 samples from Ely Farm (UK), 10 samples from MespolMedlov, A.S. (Czech Republic), and 14 samples from Wageningen University experimental farm (The Netherlands) [30, 37]. A total of 67 samples from the Karacabey field were pooled together in one matrix with the 324 external samples. The remaining 25 samples were used for validation of the laboratory scanned vis-NIR measurements. The calibration matrix set of 391 (67 + 324) was used to develop the SOC calibration model.

The calibration spectra were pretreated. Firstly, the raw spectra at both edges were trimmed to get the final wavelength range of spectra (370 to 2150 nm). Secondly, soil spectra were averaged for three and fifteen neighboring wavelengths in the ranges of 370–1000 nm and 1001–2150 nm, respectively. This was followed by maximum normalization, 1st Savitzky-Golay derivation, and smoothing with Savitzky-Golay method [4]. The pretreated spectra and the results of laboratory chemical analyses were used to develop the calibration model for SOC. PLS regression with one-leave-out cross-validation was carried out using the calibration set to develop SOC calibration model using Unscrambler 7.8 software (Camo Inc., Oslo, Norway).

The performance and accuracy of the SOC calibration model were evaluated in cross-validation and independent validation. The independent online validation was carried out using the online soil spectra of the validation set of 25 soil samples. Model performance was evaluated by means of coefficient of determination (*R* ^2^), root mean square error of prediction (RMSEP), and ratio of prediction deviation (RPD) that is standard deviation divided by RMSEP. Sample statistics of the calibration and independent validation sets for SOC model are shown in Table 2.

### 2.6. Development of SOC and NDVI Maps

Three categories of SOC maps were developed: (1) laboratory reference analyses maps (based on 92 and 25 independent validation samples), (2) laboratory and online validation maps based on 25 independent validation points, and (3) full-data points maps based on all online vis-NIR predicted points (6486). Inverse distance weighing (IDW) interpolation method was used to develop the laboratory measured, laboratory predicted, and online predicted maps of categories 1 and 2. IDW method is based on the extent of similarity of cells, while methods, such as trend fitting of a smooth surface, are defined by mathematical function. Kriging is a statistical method used in diverse application modeling. Both interpolation methods to develop different maps use ArcGis 10 (ESRI, USA) software. The advanced parameters option allowed control of the semivariogram used for Kriging.

The assessment of normality of the data comparison assumption can be divided into visual inspection and statistical tests. The simplest way to compare maps is visual inspection to look for similarities that may exist or not. However, this is not sufficient, a quantitative estimation of similarities as a more robust approach needs to be adopted. To visualize relationship between different maps, ArcGIS Geostatistical Analyst General Quantile-Quantile (Q-Q) tool was used. To compare statistical relationship between pairs of maps Kappa statistics [38], to calculate Kappa value (*κ*), were performed using SPSS (Statistical Package for the Social Sciences, IBM, USA). Before running Kappa statistics maps were rasterized to 25754 points by assigning value 3 as output cell size. The Kappa statistics were carried out for the following pairs of maps:

1. laboratory measured versus laboratory vis-NIR predicted SOC of the independent 25 samples,
2. laboratory measured versus online vis-NIR predicted SOC of the independent 25 samples,
3. laboratory measured (92 samples) versus NDVI map,
4. full-point vis-NIR predicted (6486 points) versus NDVI map.

The analyses for cases 1 and 2 were selected to make a comparison between laboratory measured and online predicted SOC maps, whereas cases-3 and 4 were selected to make a comparison between both laboratory measured and online vis-NIR predicted SOC maps with the NDVI map.

## 3. Results and Discussion

### 3.1. Model Performance in Calibration and Independent Validation

SOC model performance in cross-validation, laboratory, and online validations is provided in Table 3. Results show that SOC calibration model in cross-validation results is fairly accurate (*R* ^2^ = 0.75, RMSEP = 0.17, and RPD = 1.81). According to the classification of RPD values proposed by Viscarra-Rossel et al. [39], the performance of the SOC in cross-validation is classified as good. This finding is in coherence with earlier reports by Udelhoven et al. (with *R* ^2^ = 0.60 and RMSEP = 1.4%) [40] and by Dunn et al. (with *R* ^2^ = 0.66 and RMSEP = 2.5) [41]. However, better results were reported by Chang et al. (*R* ^2^ = 0.89, RMSEP = 6.2) [8] and Islam et al. (*R* ^2^ = 0.81, RMSEP = 3.5) [13]. The latter studies were based on analyses of dry soil samples, whereas the current study is based on the analyses of fresh soil samples. The majority of reports confirm that vis-NIR analyses based on dried and sieved soil samples result in better model performance [4, 42], compared with those with fresh soil samples, since MC affects accuracy by masking the spectral features of SOC existing in the NIR range.

**Figure 4:**
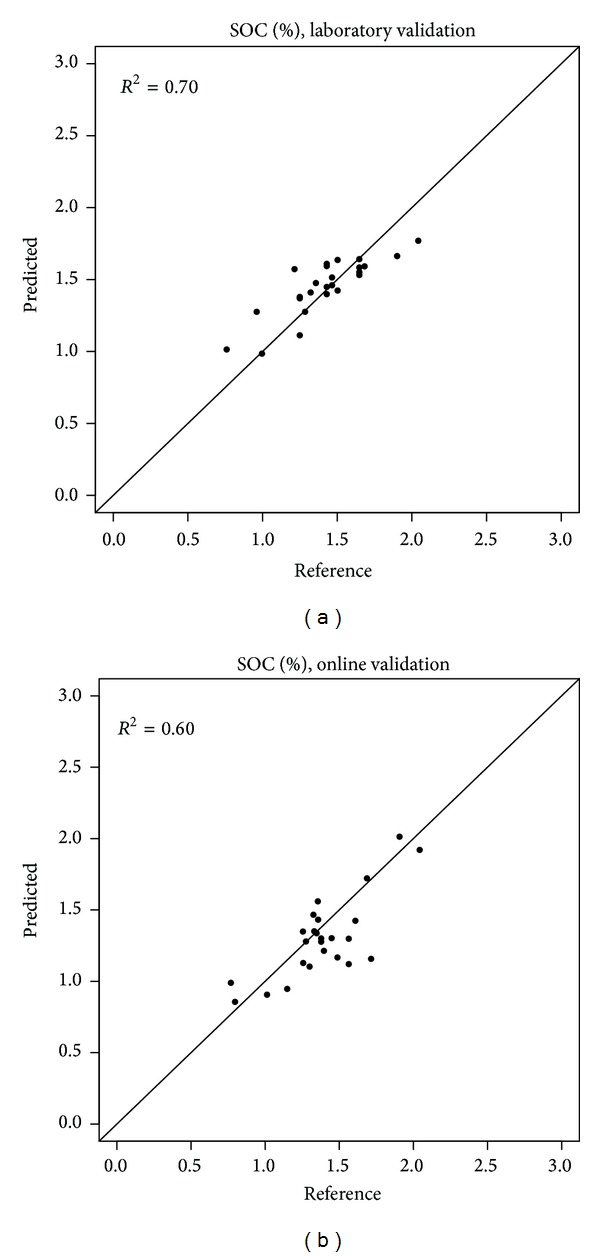
Scatter plot of predicted versus laboratory measured soil organic carbon (SOC) of the validation set (25 samples) for laboratory scanned (a) and online scanned soil spectra (b).

The performance of the vis-NIR spectroscopy for the prediction of SOC of the independent validation set under online measurement condition was not as good as that under laboratory measurement condition (Table 3). According to the classification of RPD values proposed by Viscarra-Rossel et al. [39], bother laboratory (RPD = 1.78) and online independent (RPD = 1.41) validations are classified as fair models/predictions (RPD values are between 1.4 and 1.8).  Figure 4 shows the scatter plots of measured versus predicted SOC for laboratory validation and online validation. A better match of predicted versus measured SOC can be observed for the laboratory scanned spectra as compared to the online measured spectra, using the same soil samples (e.g., 25 soil samples). A relatively low model prediction performance compared to a previous report, using the same online sensor, was found as shown in Table 3 [21]. Kuang and Mouazen [21] observed clear increases in RPD values with spiked sample number per ha. On the basis of average values of the RPD of the three studied fields, authors reported that, by an increase in spiked sample number from 1/1.5 to 3.5/4.5 per ha, an average increase in RPD of 9.1% can be expected [21]. In this study about 6.5 samples per ha were spiked (67 samples for 10 ha) in the general sample set, which rejects the assumption of the effect of low number of spiked samples on resultant accuracy. Another reason for low accuracy obtained in this work might be due to the narrow variation range of the field SOC (Table 2) [21]. However, Kuang and Mouazen [21] also claimed that higher *R* ^2^ and RPD for a larger variability dataset (larger range of concentration) can be obtained, but RMSEP would also be larger compared to a dataset with a small range of variability.

**Figure 5:**
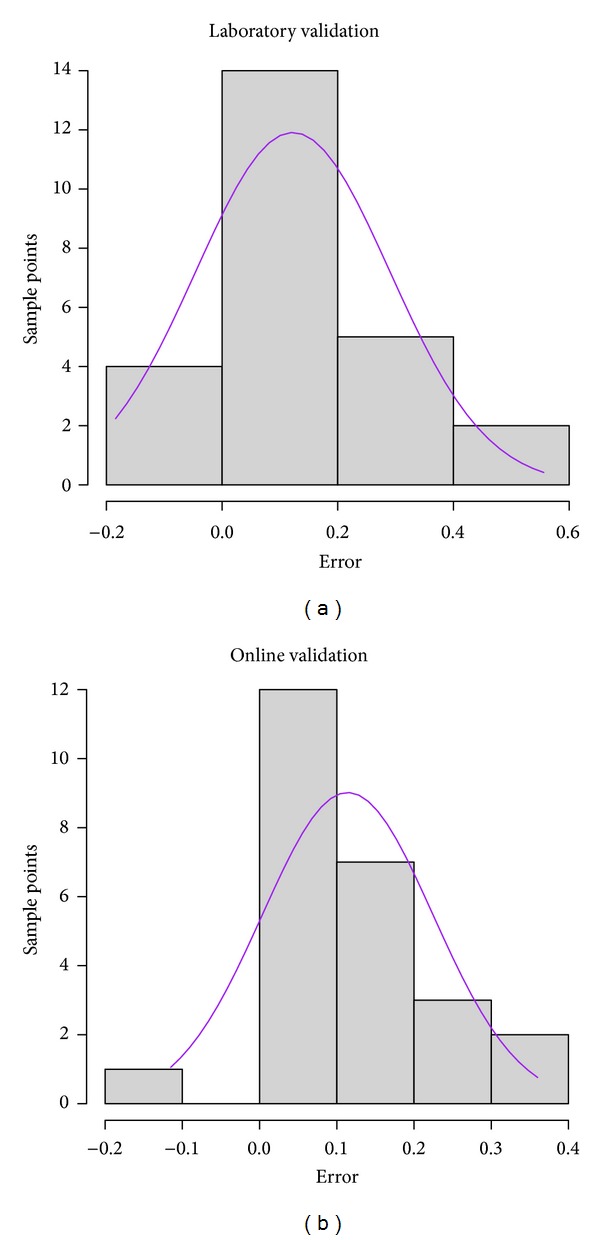
Histogram of normal distribution of error for laboratory (a) and online (b) predictions of soil organic carbon (SOC).

The histogram of normal distribution plots of error was calculated by subtracting SOC predicted from measured values using the 25 samples of the independent validation set scanned under laboratory (Figure 5(a)) and online (Figure 5(b)) conditions. These plots show overprediction of both cases, as clear skewness toward the positive range of error can be observed. More points on the negative error range are calculated for the laboratory scanning as compared to the online scanning. A further analysis of error (e.g., error of maps) is needed, as the normal distribution of error cannot provide sufficient data to support this analysis.

### 3.2. Mapping

#### 3.2.1. Comparison Maps of Laboratory and vis-NIR Maps of SOC

**Figure 6:**
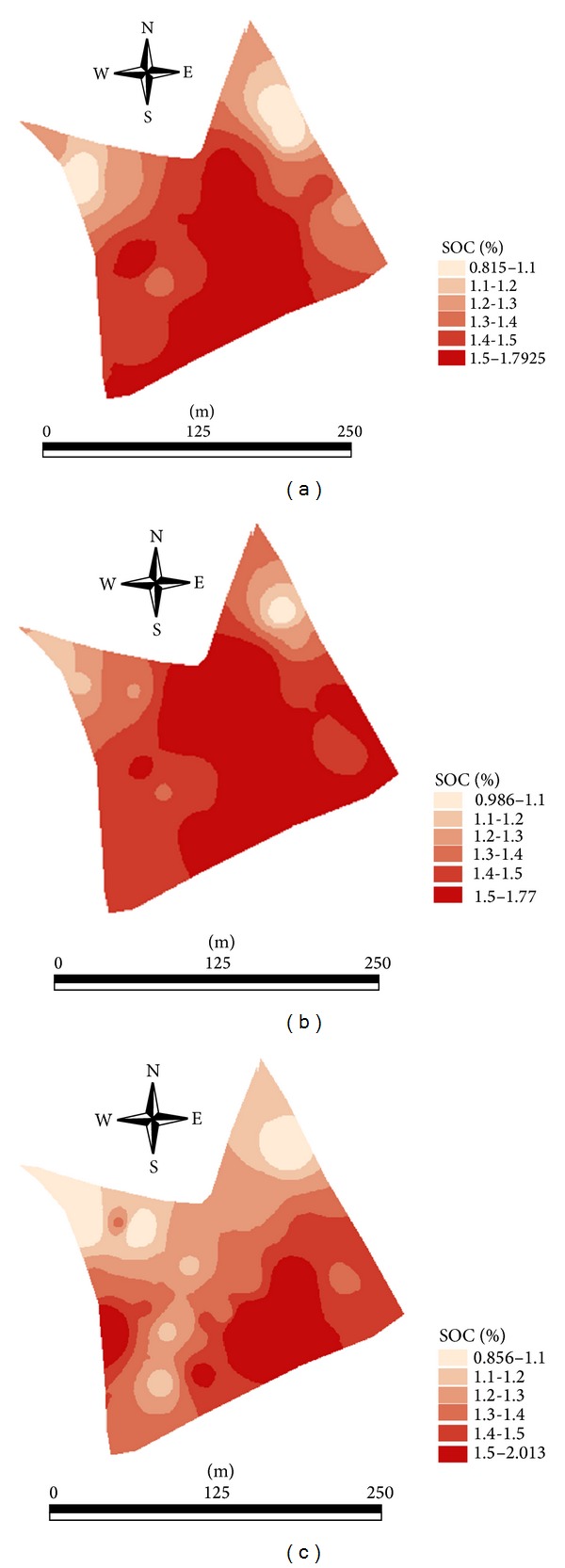
Comparison maps of soil organic carbon (SOC) between laboratory measured (a), laboratory visible and near infrared (vis-NIR) predicted (b), and online vis-NIR predicted (c), based on the 25 samples of the independent validation set maps.

Comparison maps between laboratory measured, laboratory vis-NIR predicted, and online vis-NIR predicted maps using the independent validation set of 25 samples show reasonable spatial similarity with high and low concentration zones of SOC distinguishable clearly. However, a visual comparison of these maps reveals presence of a better spatial similarity between the laboratory measurement (Figure 6(a)) and laboratory vis-NIR prediction (Figure 6(b)), as compared to online vis-NIR prediction map (Figure 6(c)).

This is as expected, because, during the online measurement, ambient conditions such as vibrations, presence of dust, stones, and roots have negative impact on accuracy [8]. These conditions affect the prediction accuracy compared to vis-NIR laboratory analyses, which is performed under controlled conditions. This result coincides with the previous reports [43]. Another source of error might be attributed to the mismatch of sample position with corresponding spectra of the 25 samples collected for validation during the online measurement, as reported earlier [4].

**Figure 7:**
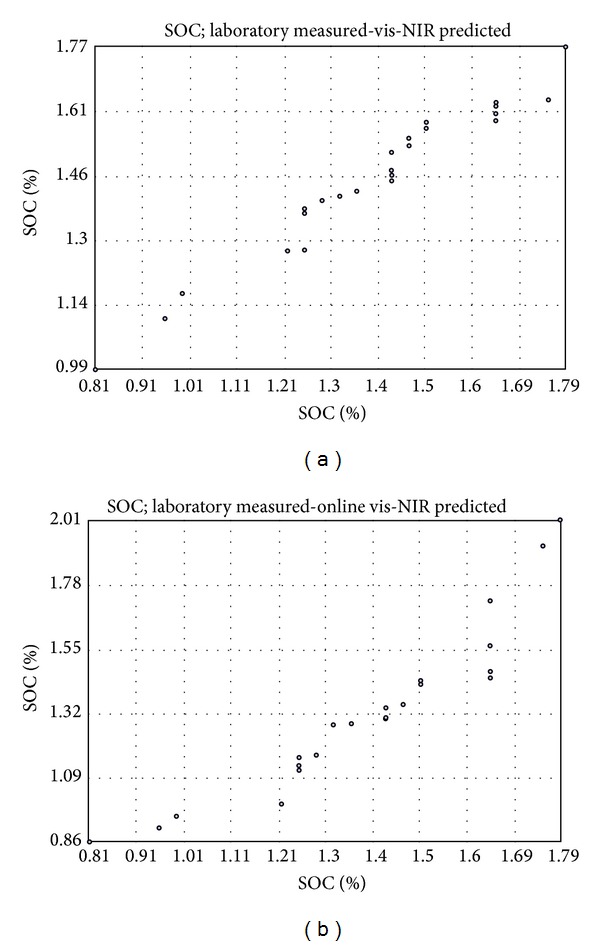
Q-Q plots between laboratory measured versus laboratory visible and near infrared (vis-NIR) predicted (a) and laboratory measured versus online vis-NIR predicted (b) soil organic carbon (SOC). The two plots were based on 25 samples of the validation set.

General Q-Q plot was used to assess the visual similarity of the distributions of datasets (Figure 7). Q-Q plots compare the quintiles of data distribution with the quintiles of standardized theoretical distribution from a specified family of distributions [44]. Q-Q plots for laboratory measured SOC versus laboratory vis-NIR predicted SOC (Figure 7(a)) based on 25 samples of the validation set show normal distribution of the datasets, indicating high similarity between the two maps. However, in Q-Q plot for laboratory measured SOC versus online vis-NIR predicted SOC (Figure 7(b)), there are two points, one at 1.65% and the other at 1.21% SOC. Both the points are vertically separated from the rest of the 25 points, indicating a deterioration of similarity with the online predicted map, caused by the ambient conditions and/or the error attributed to the mismatch of sample position and corresponding spectra of the 25 samples collected for validation during the online measurement.

Landis and Koch [45] categorized Kappa values as <0.0—no agreement, 0.00–0.20—slight agreement, 0.21–0.40—fair agreement, 0.41–0.60—moderate agreement, 0.61–0.80—substantial agreement, and 0.81–1.00—excellent agreement. The output of the Kappa test comparing laboratory measured and laboratory vis-NIR predicted SOC maps indicates higher spatial similarity between laboratory measured and laboratory vis-NIR predicted maps as compared to that between laboratory measured and online vis-NIR predicted maps (Table 4). The *κ* values of the former and the latter comparisons were 0.473 and 0.367, respectively. This is another clue to conclude that the laboratory vis-NIR predicted SOC map resembles better the corresponding laboratory measured map than the online vis-NIR predicted map.

#### 3.2.2. Similarities between SOC and NDVI Maps

**Figure 8:**
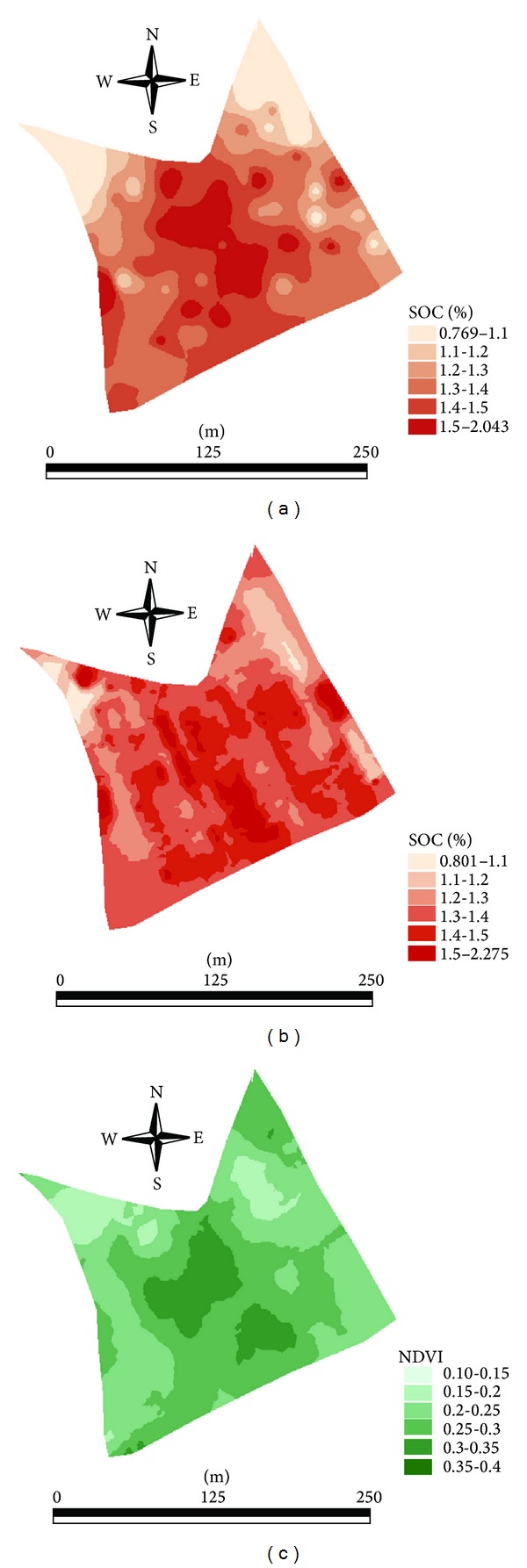
Comparison between maps of laboratory measured soil organic carbon (SOC) (92 samples) (a), online visible and near infrared (vis-NIR) predicted full-point SOC (6486 points) (b), and normalized difference vegetation index (NDVI) (c).

Comparison between laboratory measured (all 92 samples) SOC, online predicted full-point (6486 points) SOC, and NDVI maps indicates reasonable spatial similarity (Figure 8). The full-point SOC map (Figure 8(b)) shows similarity across the field area ranging from 0.801% to 2.275%.

Higher SOC concentrations are observed along the middle part of the field, whereas lower concentrations can be observed at the triangular edges and at the northern part of the field. NDVI map also shows similar spatial distribution to that of SOC, with larger values (0.30 to 0.40) at the middle part of the field as compared to the field edges, whose NDVI values are lower (0.1 to 0.2). This similarity between the online full-point map and NDVI map indicates crop response to soil fertility by producing more crop biomass at high SOC concentration zones. The low values of NDVI might be attributed to the late measurement (in May), at that time the crop turns into yellowish stage. Naser et al. [46] stated that the NDVI values decreased from anthesis to midgrain filling stage because reflectance from red band increased and reflectance from NIR band decreased.

**Figure 9:**
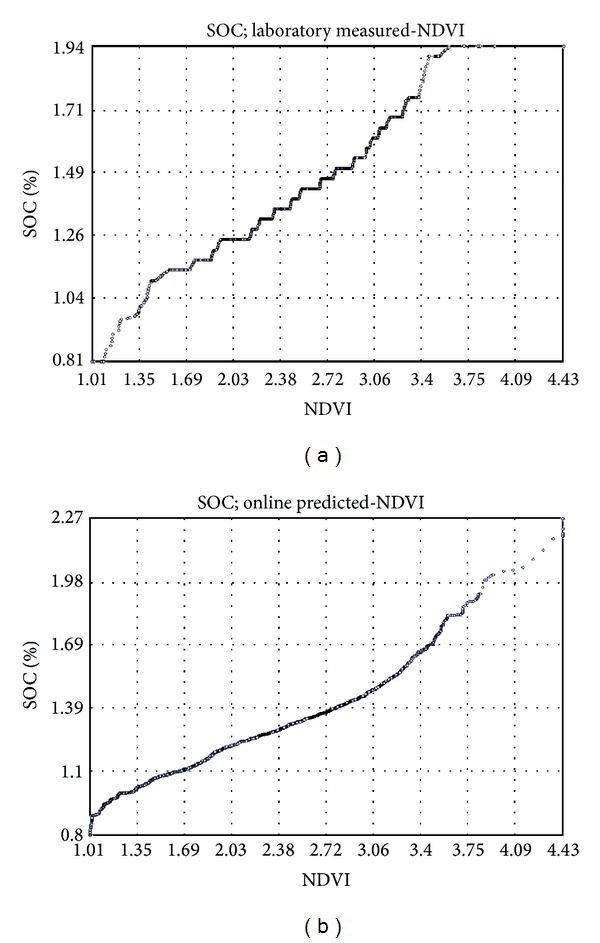
Q-Q plots between laboratory measured (92 samples) soil organic carbon (SOC) versus normalized difference vegetation index (NDVI) (a) and online vis-NIR predicted (6486 points) versus NDVI (b).

The Q-Q plot of laboratory measured (92 samples) SOC versus NDVI (Figure 9(a)) shows staircase pattern of the distribution, which means that some values are discrete from the normal distribution. However, the Q-Q plot of NDVI and online full-point SOC map (Figure 9(b)) show a straight, smooth line, confirming visual similarities to be well represented.

The output of the Kappa statistics comparing laboratory measured SOC (92 samples) and vis-NIR predicted SOC maps on the one hand and NDVI on the other hand indicates rather poor spatial similarities (Table 4). The *κ* values between laboratory measured SOC and NDVI maps and online vis-NIR predicted full-point SOC and NDVI maps were 0.20 and 0.24, respectively. This indicates larger similarity in the latter case as compared to the former case, which confirms the high sampling resolution obtained with the online sensor to be more appropriate to understand spatial distribution of crop growth as related to SOC distribution.

## 4. Conclusions

This study was undertaken to map the spatial variability in soil organic carbon (SOC) in one clay field using an online visible and near infrared (vis-NIR) soil sensor, which is capable of providing high resolution data. It also aimed to evaluate correlations between SOC measured with laboratory methods and online sensor on one hand with crop normalized difference vegetation index (NDVI) of wheat, measured with a proximal sensor, on the other hand. The obtained results led to the following conclusions.

1. The SOC calibration model in cross-validation resulted in moderate accuracy (*R* ^2^ = 0.75, RMSEP = 0.17%, and RPD = 1.81). The independent validation resulted in a better performance for laboratory scanned spectra (*R* ^2^ = 0.70, RMSEP = 0.15%, and RPD = 1.78) as compared to the online measurement (*R* ^2^ = 0.60, RMSEP = 0.20%, and RPD = 1.41).
2. Similarity between SOC measured and predicted maps was evaluated with the Kappa statistics, which indicated better similarity between laboratory measured and laboratory vis-NIR predicted maps (*κ* = 0.473) than that between laboratory measured and online vis-NIR predicted maps (*κ* = 0.367).
3. Similarity between laboratory measured SOC (92 points) and NDVI maps evaluated with the Kappa test showed rather poor similarity (*κ* = 0.203). An improved similarity was found between the online vis-NIR prediction of SOC (6486 points) and the NDVI map (*κ* = 0.259), indicating a better link between crop development and soil fertility (e.g., SOC), measured with high sampling resolution using the online soil sensor.

A future research should explore correlations between other soil fertility parameters (e.g., pH, cation exchange capacity (CEC), and NDVI). This may also include correlations not only with crop growth indicated as NDVI, but also with crop yield, as the latter is directly linked with farm production efficiency and food security issues in particular.

## Figures and Tables

**Table 1:** Information about the Karacabey field in Bursa, Turkey, where soil and crop measurements were taken in the spring and summer of 2013.

Area, Ha	Crop	Sample Number	Texture class	Sand %	Silt %	Clay %	MC, %			
							Min.	Max.	Mean	SD
10	Wheat	92	Clay	26.6	30.4	43	14.9	45.4	22.4	4.8

MC: moisture content.

**Table 2:** Sample statistics of laboratory and online measured SOC (%) of the calibration and independent validation sets.

Karacabey field	Sample number	Min, %	Max, %	Mean, %	SD, %
All field samples	92	0.81	1.93	1.41	0.22
Cross-validation set	391	0.79	2.64	1.41	0.31
Laboratory validation set	25	0.98	1.66	1.44	0.17
Online validation set	25	0.85	2.01	1.29	0.28

SD = standard deviation.

**Table 3:** Summary of SOC model performance in cross-validation, laboratory and online validations.

Karacabey field	*R* ^2^	RMSEP, %	RPD	Intercept	Slope
Cross-validation	0.75	0.17	1.81	0.03	0.97
Laboratory validation	0.70	0.15	1.78	0.61	0.59
Online validation	0.60	0.20	1.41	0.25	0.76

**Table 4:** Results of Kappa test comparing symmetric measures of maps of soil organic carbon (SOC) and normalized difference vegetation index (NDVI).

Pairs	Symmetric measures		*κ*
	Asymp. Std. Error^a^	Approx. *T* ^b^	
Laboratory measured-laboratory vis-NIR predicted SOC (25 samples)	0.004	159.722	0.473
Laboratory measured-online vis-NIR predicted SOC (25 samples)	0.004	118.344	0.367
Laboratory measured SOC (92 samples)-NDVI	0.004	71.143	0.203
Online vis-NIR predicted SOC (6486 points)-NDVI	0.004	77.392	0.259

*κ*: Kappa.

a: not assuming the null hypothesis.

b: using the asymptotic standard error assuming the null hypothesis.
